# Transcultural Adaptation and Validation of the Spanish Bristol Foot Score (BFS-S)

**DOI:** 10.14336/AD.2017.1215

**Published:** 2018-10-01

**Authors:** Emmanuel Navarro-Flores, Marta Elena Losa-Iglesias, Ricardo Becerro-de-Bengoa-Vallejo, Daniel Lopez-Lopez, Juan Manuel Vilar-Fernandez, Patricia Palomo-Lopez, Cesar Calvo-Lobo

**Affiliations:** ^1^Faculty of Medicine, Universidad Miguel Hernandez de Elche, and Department of Nursing and Podiatry, University of Valencia, Spain; ^2^Faculty of Health Sciences, Universidad Rey Juan Carlos, Spain; ^3^School of Nursing, Physiotherapy and Podiatry, Universidad Complutense de Madrid, Spain; ^4^Research, Health and Podiatry Unit, Department of Health Sciences, Faculty of Nursing and Podiatry, Universidade da Coruna, Spain; ^5^Modeling, Optimization and Statistical Inference Research Group, Universidade da Coruna, Spain; ^6^University Center of Plasencia, Universidad de Extremadura, Spain; ^7^Nursing and Physical Therapy Department, Institute of Biomedicine (IBIOMED), Universidad de Leon, Ponferrada, Leon, Spain

**Keywords:** foot, quality of life, health impact assessment, validation studies

## Abstract

The Bristol Foot Score is considered an instrument for measuring the impact of foot problems and pain. It was developed and validated in United Kingdom. Therefore, this aim was to perform the transcultural adaptation and validation of the Spanish version. The recommended forward/backward translation protocol was applied for the procedure of translation, transcultural adaptation and validation to Spain. Considering each domain and question, internal consistency and reliability were analyzed through the Crombach alpha (α) and intraclass correlation coefficient (ICC) with a 95% confidence interval (95% CI). A very good internal consistency was shown for the 3 domains: concern and pain showed a Cronbach of 0.896, footwear and general foot health of 0.790, mobility 0.887. Each question had a very good test-retest reliability, ranged from 0.721 to 0.963 with no systematic differences (P>0.05) in each question of the Spanish Bristol Foot Score (BFS-S) questionnaire. The test-retest reliability was excellent (ICC 95%): concern and foot pain 0.950 (0.913-0971); footwear and general foot health 0.914 (0.851-0.950), mobility 0.973 (0.953-0.984) and there were no sistematic differences in any domain (P > 0.05). The BFS-S was shown to be a valid and reliable tool with an acceptable use in the Spanish population.

Worldwide, clinimetric tools such as the Foot Health Status Questionnaire (FHSQ), Foot Function index (FFI) as well as Manchester Foot Pain and Disability Index (MFPDI) were validated and translated for assessing the quality of life related to patient’s foot health [1-4]. Approximately, foot pain and disorders were presented in 25% of the adult population [5]. Up to 8% of musculoskeletal pain consultations by general practitioners were related to foot and ankle conditions [6]. Indeed, foot pain may increase this prevalence in older adults with specific foot conditions being associated to higher disability [7]. In addition, the worst quality of life related to the foot health may be associated to the risk increase of fall [8,9].

The Bristol Foot Score (BFS) may be considered as a self-reported health questionnaire with 15 items for measuring the impact of foot problems such as concern and pain (7 items), footwear and general foot health (4 items), and patient mobility (3 items). The BFS was developed and validated in the United Kingdom with a high reliability (Cronbach α = 0.90) [10]. This questionnaire is sensitive to change after toenail surgery. Nevertheless, a poor level of concordance was reported between the BFS and the Chiropody Assessment Criteria Score [10,11]. Consequently, the BFS may reflect patients’ perceptions of their own foot health and may be useful for assessing the efficacy after interventions and establishing foot health within populations [10]. Despite the domains of the FHSQ (foot pain, foot function, footwear, and general foot health), FFI (pain, disability and activity limitation) and MFPDI (foot pain and function) may be considered similar tools validated and translated into Spanish [1-4], specifically the BFS adds new domains such as the patient mobility [10].

Considering the BFS domains, 3 underlying factors were considered. First factor, concerns about feet and pain was shown to be the most powerful to predict the 50% of the set of 15 responses. Second and third factors, footwear and general foot health as well as mobility were reported to predict the 10% and 9% of the variance, respectively [10]. Nevertheless, transcultural adaptation, contruct validity and reliability should be carried out following guideliness in order to preservate the crosscultural measurement properties [3,12-14]. To date, the BFS has not been adapted or validated to Spanish language [10,15]. Therefore, this study aim was to perform the transcultural adaptation and validation of the Spanish BFS version (BFS-S).

## MATERIALS AND METHODS

### Study design

A cross-sectional descriptive study was carried out between june and september 2017, following The Strengthening the Reporting of Observational Studies in Epidemiology (STROBE) statement and checklist [16]. Transcultural adaptation and validation was performed using the BFS as a clinimetric tool [10].

### Ethical statements

The Ethics Committee approval was obtained from the University of La Coruña. Furthermore, informed consent was obtained from all subjects. The Helsinki Declaration, Organic Low of Protection Data (15/1999) and ethical standards in human experimentation were respected.

### Participants

A total sample of 53 participants with a mean ± SD (range) of 49.55 ± 16.17 (23-78) years, 69.26 ± 11.92 (47-98) kg, 168 ± 0.08 (151-189) cm and 24.20±3.37 (17.68-35.54) kg/cm^2^ was recruited from podiatry and physiotherapy clinical centers. Inclusion criteria comprised participants with foot pain for at least the past 3 months. Exclusion criteria included psychiatric or cognitive disorders in the medical record, refusal to give consent form and the inability to following the instructions necessary to carry out the present investigation [1-4,10].

### Translation procedure

The recommended forward/backward translation protocol was applied for the procedure of translation, transcultural adaptation and validation from United Kingdom to Spain [2,3,12-14]. The translation procedure was conducted according to the recommended international guidelines [12,17].

First, the author of the original questionnaire (SB) was contacted in order to carried out this translation [10]. Second, forward translation was performed by two independent bilingual Spanish translators. Third, the reconciliation in the forward translations was performed and written with each translator separately. Fourth, the reconciled forward translated version of the BFS-S was translated back to Spanish by 4 authors (ENF, DLL, PPL and CCL), 3 podiatrists and 1 physiotherapist PhD university professors. Fifth, the translated version was compared with the original version to be sure about conceptual equivalence of the translation, discrepancy or unclear terms. Sixth, the harmonization was carried out by an expert panel formed by 6 authors (ENF, DLL, PPL, CCL, MELI and RBBV), 5 podiatrists and 1 physiotherpist PhD university professors, in order to be agreeing about the translation. Seventh, cognitive interviews were carried out in physiotherapy and podiatry centers in order to provide validity and avoid potential errors [17]. Finally, the proofread version of the BFS-S was composed by a Likert scale to improve administration and psychometric properties [2,10].

### Test-retest reliability and sample size

Test-retest was performed by the following link: https://docs.google.com/forms/d/e/1FAIpQLSfMGyHjbZf75C23562HVZlfoUhpPA_1SozoN_UvzU9p6dZgHw/viewform. Furthermore, the sociodemographic data (age, sex, profession and study degree), comorbidities (diabetes, peripheral vascular disease, rheumatism, psoriasis, and osteoarthritis), lifestyle (sedentary or active) and foot conditions were self-reported in this link. Participants with foot conditions were recruited from podiatry and physiotherapy clinical centers where universitary students carried out their practices. A pilot study was conducted in order to establish the linguistic comprehension of the BFS-S. Considering a correlation with an ICC of 0.40 and a 95% confidence interval (CI) for a two-tailed test, an error α of 0.05 and a desired analysis power of 80% (error β = 20%), a final sample size of 53 paticipants was obtained. The sample was heterogeneous in order to test this questionnaire for multiple and variated foot conditions [2]. The questions and domains (concern and pain; footwear and general foot health; and mobility) scores of the BFS-S were collected [10]. All patients were able to complete the questionnaire by themselves and the time employed in filling it out was approximately about 5 minutes.

**Table 1:** Socio-demographic characteristics of the sample population.

	Total group Mean ± SD Range N = 53	Men Mean ± SD Range N = 23	Women Mean ± SD Range N = 30	P Value
Age, years	49.55±16.17 (23-78)	54.33±15.32 (47.86-60.79)	45.58±16.01 (39.49- 51.66)	0.004
Weight (kg)	69.26±11.92 (47-98)	72.20±9.11 (73.35-81.04)	62.68±9.84 (58.93-66.42)	0.747
Height (cm)	168±0.08 (151-189)	1.74±0.7 (1.70-1.77)	164±0.05 (161-166)	0.756
BMI (kg/m2)	24.20±3.37 (17.68-35.54)	25.42±0.07 (25.39-25.44)	23.18±3.69 (21.77-24.58)	0.082

*Abbreviations*: BMI, body mass index; SD, standard deviation. In all the analyses, P < .01 (with a 99-confidence interval) was considered statistically significant. P-values are from Independent student t-test.

### Statistical analysis

All variables were examined for normality of distribution using the Kolmogorov-Smirnov test, and data were considered normally distributed if *P* > 0.05. Independent Student t-tests were performed to find if differences are statistically significative when showing a normal distribution. Measurements which were not normally distributed were tested using non-parametric Wilcoxon signed-rank test. Considering each domain and question, internal consistency and reliability were analyzed through the Crombach alpha (α) with 0 indicating no internal consistency and 1 corresponding to perfect internal consistency and intraclass correlation coefficient (ICC) with a 95% confidence interval (95% CI). To interpret ICC values, we used benchmarks as proposed by Landis and Koch [18]: 0.20 or less, slight agreement; 0.21 to 0.40, fair; 0.41 to 0.60, moderate; 0.61 to 0.80, substantial; and 0.81 or greater, almost perfect. For the statistical analysis, a two-way random effects model (2.1), single measures, absolute agreement, and ICC were used to express reliability. In addition, paired samples t-test was applied to test systematic differences between test and retest. The use of coefficient of variation (CV) values has been the most common approach previously to examine variability between tests, and in the current study, a %CV for method error was calculated as follows: CV = 100 × (2 × (SDd/√2)/(X1 + X2) [19]. SDd represents the standard deviation of the differences between the two tests, and X_1_ and X_2_ represent the two-test means, respectively. The 95% limits of agreement statistics (LoA) were also calculated for the absolute comparison of parameters and express the degree of error proportional to the mean; the statistics were calculated using the methods described by Bland and Altman [20] and if the differences between the measurements tend to agree, the result will be close to zero. In addition, standard errors of measurement (SEM) were calculated to measure the range of error of each gait parameter. SEM is a quantitative expression of the range of error that can occur whenever the same participant repeats certain tests. In addition, SEM values were calculated from the ICCs and SDs for each session, using the higher of the 2 SD measurements to determine the range of error attributed between sessions. SEM were calculated according to the formula SEM = SD × sqrt (1 - ICC). Similarly, and for convenience of interpretation, the percent error of the SEM (SEM%) was calculated as the SEM divided by the mean per 100 and provided an estimate of the inherent error or variability normalized to the mean (SEM % = SEM/mean*100 %) [20]. In addition, to determine the smallest amount of change that is real and beyond the bound of measurement error, minimum detectable changes (MDC) were calculated at a confidence level of 95%: MDC values, which reflect the magnitude of change necessary to provide confidence that a change is not be the result of random variation or measurement error, were calculated as follows [21]: MDC = √2 × 1.96 × SEM. Furthermore, Bland and Altman plots were analyzed to evaluate agreement and heteroscedasticity [20]. Each measure was evaluated for homoscedasticity with Breusch-Pagan test for heteroscedasticity (P < 0.05) in a linear regression model [22]. A P value < 0.05 with a confidence interval of 95% was considered statistically significant for all tests (SPSS for Windows, version 20.0; SPSS Inc., Chicago, Illinois).

**Table 2:** Results of reliability, test-retest of the Spanish Bristol Foot Score (BFS-S) questionnaire according to each question.

	TEST (n=53) Mean ± SD (CI 95%)	RETEST (n=53) Mean ± SD (CI 95%)	ICC (CI 95%)	P- value	SEM	%CV	SEM%	LoA Mean diference (limits)	MDC	P-value Breusch-Pagan
Question 1. Do problems with your feet affect whether you go out of the house to visit family or friends?	1.60±0.92 (1.34-1.85)	1.56±0.86 (1.32-1.80)	0.963 (0.936-0.79)	0.419	0.005	1.684	0.321	0.038 (-0.324-0.999)	0.014	0.103
Question 2. Do problems with your feet affect whether you walk to the shops?	1.62±0.90 (1.37-1.87)	1.67±0.91 (1.42-11.93)	0.959 (0.928-0.976)	0.261	0.008	2.424	0.494	-0.057 (-0.348-1.073)	0.023	0.239
Question 3. Do problems with your feet affect you when standing still?	1.67±0.97 (1.41-1.94)	1.73 ±.092 (1.48-1.99)	0.962 (0.935-0.978)	0.261	0.008	2.344	0.457	-0.057 (-0.348-1.073)	0.022	0.001
Question 4. Do problems with your feet affect you when walking on bumpy or stony ground?	2.18±1.05 (1.98-2.48)	2.15 ±.1.02 (1.86-2.43)	0.944 (0.903-0.968)	0.569	0.006	1.230	0.289	0.038 (-0.460-1.417)	0.017	0.007
Question 5. Over the last two weeks how painful have your feet been?	2.16±1.29 (1.81-2.52)	2.13±1.27 (1.78-2.48)	0.897 (0.822-0.941)	0.727	0.024	2.417	1.107	-0.075 (-1.095-3.377)	0.068	0.002
Question 6. Over the last two weeks, how often have you felt this way about your feet? "I have felt conscious of my feet".	2.81±1.75 (2.32-3.29)	2.64±1.71 (2.16-3.11)	0.918 (0.857-0.953)	0.201	0.026	3.402	0.949	0.132 (-0.884-2.725)	0.072	0.091
Question 7. Over the last two weeks, how often have you felt this way about your feet? "I have felt fed up about my feet".	2.45±1.68 (1.98-2.91)	2.35±1.69 (1.89-2.82)	0.950 (0.913-0.971)	0.358	0.015	2.773	0.622	0.094 (-0.711-2.192)	0.041	0.010
Question 8. Over the last two weeks, how often have you felt this way about your feet? "I have felt worried that my feet will get worse in the future".	2.54±1.61 (1.79-2.69)	2.13±1.56 (1.69-2.56)	0.933 (0.883-0.961)	0.308	0.021	3.657	0.950	0.113 (-0.768-2.369)	0.058	0.926
Question 9. Over the last two weeks, have you felt this way about your feet? "I have felt my feet are not really part of me".	1.37±0.62 (1.20-1.55)	1.30±0.57 (1.14-1.46)	0.886 (0.803-0.934)	0.159	0.018	3.984	1.356	0.075 (-0.369-1.139)	0.050	0.163
Question 10. Because of your feet have you had problems sleeping, in the last two weeks?	1.24±0.75 (1.03-1.45)	1.28±0.76 (1.07-1.49)	0.984 (0.972-0.991)	0.159	0.003	2.111	0.271	-0.038 (-0.185-0.569)	0.009	0.055
Question 11. In the last two weeks have you been able to put your everyday shoes on easily.	1.60±0.83 (1.37-1.83)	1.79±1.02 (1.50-2.07)	0.701 (0.481-0.827)	0.133	0.074	7.857	4.336	-0.189 (-0.864-2.664)	0.204	0.001
Question 12. Over the last two weeks how often have you been able to wear any shoes you liked.	1.94±1.44 (1.15-2.34)	2.15±1.59 (1.71-2.59)	0.888 (0.806-0.935)	0.125	0.050	7.169	2.429	-0.208 (-0.929-2.865)	0.138	0.001
Question 13. If you could afford any shoes you wanted, how easily could you find new shoes that fit comfortably?	2.16±0.87 (1.92-2.40)	2.11±0.84 (1.87-2.34)	0.860 (0.758-0.919)	0.497	0.015	1.869	0.696	0.057 (-0.578-1.781)	0.041	0.072
Question 14. In general, would you say your foot health is:	2.64±1.19 (2.37-2.97)	2.58±1.18 (2.25-2.91)	0.939 (0.895-0.965)	0.472	0.010	1.532	0.376	0.057 (-0.546-1.684)	0.027	0.917
Question 15. Would you say your general health is:	2.62±0.94 (2.36-2.88)	2.64±0.85 (2.40-2.87)	0.957 (0.925-0.975)	0.709	0.003	0.507	0.104	-0.019 (-0.352-1.085)	0.008	0.635

*Abbreviations:* SD, Standard Desviation; CI 95%, confidence interval 95%; ICC, Intraclas Correlation Index. P value from Wilcoxon Signed-Rank Test; SEM, standard error of measurement; %CV, coefficient of variation; SEM%, percent error of the SEM; LoA, 95% limits of agreement statistics; MDC = minimum detectable change; P value from Breusch-Pagan test for heteroskedasticity

## RESULTS

### Translation

The forward translations were performed with only minor discrepancies and a good agreement was observed between the 2 versions. The back translations between BFS and BFS-S were similar in many of the items. Cognitive interviews showed good understanding and comprehension of the BFS-S.

**Table 3:** Results of reliability, test-retest of the Spanish Bristol Foot Score (BFS-S) questionnaire according to each domain.

DOMAIN	Test Mean ± SD (CI 95%)	Retest Mean ± SD (CI 95%)	ICC (CI 95%)	P-value	SEM	%CV	SEM %	LoA Mean diference (limits)	MDC	P-value Breusch-Pagan
Concern and pain	13.69±7.81 (11.54-15.85)	13.56±7.14 (11.59-15.53)	0.950 (0.913-0971)	0.945	0.021	0.685	0.153	0.132 (-3.151-9.714)	0.058	0.020
Footwear and general foot health	8.35±3.51 (7.38-9.32)	8.64±3.63 (7.63-9.64)	0.914 (0.851-0.950)	0.487	0.059	2.354	0.691	-0.283 (-1.933-5.959)	0.163	0.002
Mobility	5.43 ±2.64 (4.70-6.16)	5.56±2.59 (4.85-6.28)	0.973 (0.953- 0.984)	0.357	0.015	1.698	0.282	-0.132 (-0.821-2.533)	0.043	0.041

*Abbreviations*: SD, Standard Desviation; CI 95%, confidence interval 95%; ICC, Intraclas Correlation Index. P value from Wilcoxon Signed-Rank Test; SEM, standard error of measurement; %CV, coefficient of variation; SEM%, percent error of the SEM; LoA, 95% limits of agreement statistics; MDC = minimum detectable change; P value from Breusch-Pagan test for heteroskedasticity

### Validation and reliability

The sociodemographic data, such as age, weight, height, and BMI, were shown in table 1. All of the demographic variables presented a normal distribution (P > 0.05) and all items and domains presented a no normal distribution (P < 0.05). Tables 2 and 3 show the test and retest means, ICC, P-value for non-parametric test, SEM, %CV, SEM%, MDC and P-values from Breusch-Pagan test for heteroscedasticity. Wilcoxon Signed-Rank test demonstrated no systematic differences between test and retest for any ítem and domain (P > 0.05), shown in table 2 and 3, respectively. Calculated between-test variabilities (%CV) for each ítem are shown in table 2 ranged from 1.230 to 3.984, except for ítem 11 and 12 with a %CV of 7.857 and 7.161, respectivley. %CV for each domain is presented in table 3, ranged from 0.685 to 2.354. The MDC values for each item, shown in table 2, ranged from 0.008 to 0.204 and each domain, table 3, ranged from 0.043 to 0.101. The SEM% values for each item, shown in table 2, ranged from 0.104 to 4.366 and each domain, table 3, ranged from 0.132 to 0.691.

**Figure 1.**
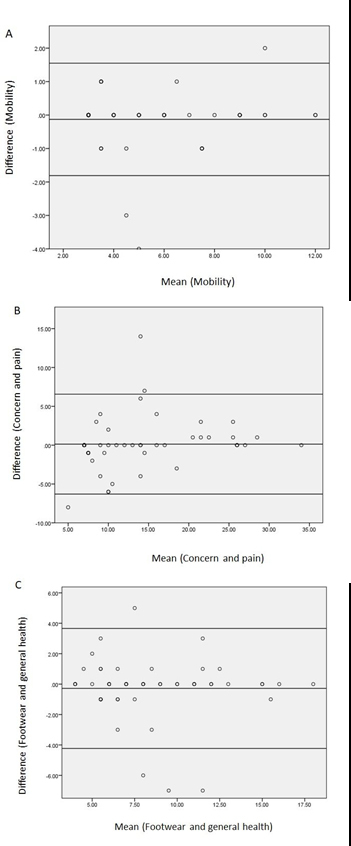
Bland-Altman plot showing the agreement between test and retest for the mobility (A), concern and pain (B), and footwear and general health (C) domains.

Results of reliability, test-retest and systematic differences of the BFS-S questionnaire by questions and domains are shown in table 2 and 3, respectively. A very good internal consistency was shown for the three domains: concern and pain showed a Cronbach of 0.896, the domain footwear and general foot health of 0.790 and domain mobility 0.887; and retest reliability was shown for each domain: concern and pain (α = 0.896; ICC = 0.950 [95% CI = 0.913 - 0971]), footwear and general foot health (α = 0.790; ICC = 0.914 [95% CI = 0.851-0950]), and mobility (α = 0.887; ICC = 0.953 [95% CI = 0.953- 0.984]). The test-retest reliability was excellent (ICC 95%): concern and foot pain 0.950 (0.913-0971); footwear and general foot health 0.914 (0.851-0.950) and mobility 0.973 (0.953-0.984) and there were no sistematic differences in any domain (P > 0.05). For total score, statistically significant differences were not shown for the mean (SD) difference between test and retest (27.49 ± 13.18 [95% CI = 23.85-21.12] points; 27.77 ± 12.37 [95% CI = 24.36-31.17] points; P = 0.658). Bland and Altman plots visual distributions did not show statistically significant or clinically relevant differences from test to retest (Fig. 1).

## DISCUSSION

Considering international recommended guidelines [12,17], The BFS-S may be used as a valid and reliable tool for measuring the self-reported health impact of foot problems such as concern and pain, footwear and general foot health, and patient mobility in the Spanish population. The original BFS was validated in the Podiatry Department at the United Bristol Healthcare National Health Service Trust with a high reliability and sensitivity to change after clinical interventions [10,11].

Previously, Spanish transcultural adaptation and validation of foot health related questionnaires were carried out with similar results [3,4]. The Spanish version of the FFI (FFI-Sp) was valid and reliable tool with a very good internal consistency for evaluating pain (0.95) and disability (0.96) of the foot [4]. Furthermore, the Spanish MFPDI version was a robust measurement tool with 3 domains such as foot pain, function and appearance due to an adequate Rasch model, excellent reliability and unidimensionality were provided [3].

To the authors' knowledge, this Spanish version may be considered as the first validation and transcultural adaptation of the original BFS. Furthermore, the BFS-S provided similar psychometric properties compared to the Spanish version of the FHSQ. An appropriated construct validity with moderate-to-high domains correlations was shown for the Spanish FHSQ (α = ≥0.739) and BFS-S (α = ≥0.790). Test-retest reliability was shown to be satisfactory for both Spanish FHSQ (ICC > 0.932) and BFS-S (ICC > 0.914) [23]. Comparing the domains from the section one of the FHSQ and the BFS, similar subscales were evaluated [1,10]. Nevertheless, the section two of the FHSQ assessed general health, physical activity, social capacity and vigour [1,24], while the BFS provided a new key domain evaluation for mobility [10].

The result generalizations of this study should be interpreted with caution due to a non-randomized consecutive sampling method was used. This study weakness may influence the participants' behavior and the procedure results in a biased sample of the domains under study [25]. The major strengths of this study comprised the first novel validation and transcultural adaptation of the BFS, as well as the possibility to evaluate the quality of life related to patient’s foot health and mobility into Spanish [10]. Furthermore, the clinical application of this questionnaire comprised the quality of life related to foot health evaluation through a new validated and reliable tool in the Spanish adult and older adult populations regarding the most common foot conditions such as metatarsalgia, hallux valgus, hallux rigidus, lesser toe deformities, hyperkeratosis, nails disorders or plantar heel pain [26].

Finally, possible limitations should be considered regarding this study. First, the BFS-S was carried out from podiatry and physiotherapy clinical centers where universitary students carried out their practices, while the original BFS was developed from a podiatry department of the healthcare national service [10]. Second, test-retest was performed through a link in the present study, while the original BFS and other Spanish validated scales were developed by face to face self-reporting of the patient [3,4,10]. Finally, age distributions such as children were not considered in this version validation, while other scales such as the Oxford ankle foot questionnaire (OxAFQ) translation was validated from 5 to 16 years old [27]. Despite it may not influence the results of transcultural adaptation and validation, there were statistically significant age differences between men and women.

### Conclusion

The BFS-S was shown to be a valid and reliable tool with an acceptable use in the Spanish population and may be used for total or each domain scores, such as concern and pain, footwear and general foot health, and patient mobility.
